# Pneumonia after Major Cancer Surgery: Temporal Trends and Patterns of Care

**DOI:** 10.1155/2016/6019416

**Published:** 2016-05-31

**Authors:** Vincent Q. Trinh, Praful Ravi, Abd-El-Rahman M. Abd-El-Barr, Jay K. Jhaveri, Mai-Kim Gervais, Christian P. Meyer, Julian Hanske, Jesse D. Sammon, Quoc-Dien Trinh

**Affiliations:** ^1^Departement of Pathology and Cellular Biology, University of Montreal Health Center, Montreal, QC, Canada H2X 0A9; ^2^Center for Surgery and Public Health, Brigham and Women's Hospital, Harvard Medical School, Boston, MA 02115, USA; ^3^Center for Outcomes Research and Analytics, Henry Ford Health System, Detroit, MI 48202, USA; ^4^Division of General Surgery, University of Montreal Health Center, Montreal, QC, Canada H2X 0A9; ^5^Department of Urology, University Medical Center Hamburg-Eppendorf, 20246 Hamburg, Germany; ^6^Department of Urology, Marien Hospital, Ruhr-University Bochum, 40479 Herne, Germany

## Abstract

*Rationale.* Pneumonia is a leading cause of postoperative complication.* Objective.* To examine trends, factors, and mortality of postoperative pneumonia following major cancer surgery (MCS).* Methods.* From 1999 to 2009, patients undergoing major forms of MCS were identified using the Nationwide Inpatient Sample (NIS), a Healthcare Cost and Utilization Project (HCUP) subset, resulting in weighted 2,508,916 patients.* Measurements.* Determinants were examined using logistic regression analysis adjusted for clustering using generalized estimating equations.* Results.* From 1999 to 2009, 87,867 patients experienced pneumonia following MCS and prevalence increased by 29.7%. The estimated annual percent change (EAPC) of mortality after MCS was −2.4% (95% CI: −2.9 to −2.0, *P* < 0.001); the EAPC of mortality associated with pneumonia after MCS was −2.2% (95% CI: −3.6 to 0.9, *P* = 0.01). Characteristics associated with higher odds of pneumonia included older age, male, comorbidities, nonprivate insurance, lower income, hospital volume, urban, Northeast region, and nonteaching status. Pneumonia conferred a 6.3-fold higher odd of mortality.* Conclusions.* Increasing prevalence of pneumonia after MCS, associated with stable mortality rates, may result from either increased diagnosis or more stringent coding. We identified characteristics associated with pneumonia after MCS which could help identify at-risk patients in order to reduce pneumonia after MCS, as it greatly increases the odds of mortality.

## 1. Introduction

Postoperative pneumonia is an important cause of morbidity and mortality and represents an important financial burden of $10.5 billion per year [1]. Patients undergoing surgery, especially complex procedures, are at a greater risk due to intubation, postsurgical atelectasis, and long hospital stays exposing them to hospital-acquired pathogens [2]. It has been estimated that approximately one out of four deaths within six days of surgery is due to its complications [3]. Furthermore, in the context of cancer surgeries, it has been suggested that certain procedures, such as lung resection, might carry a higher risk of mortality [4, 5]. Although the last two decades have seen the development of guidelines in order to standardize and improve diagnosis and treatment of hospital-acquired pneumonia [6, 7], few studies of trends have analyzed these events in the surgical context. Previous population-based studies have been restricted to admissions for pneumonia or only to limited subsets of cancer surgeries.

Based on these considerations, we performed a population-level assessment of postoperative pneumonia following major cancer surgery (MCS) for eight solid cancers. We analyzed temporal trends of postoperative pneumonia in these patients. Moreover, we identified patient and structural characteristics that are associated with pneumonia after MCS. Finally, we tested the relationship between pneumonia and in-hospital mortality. Analyzing each of the 8 procedures separately allowed us to compare their individual effects amongst each other.

## 2. Patients and Methods 

### 2.1. Data Source

Relying on the Nationwide Inpatient Sample (NIS), hospital discharges in the United States between January 1, 1999, and December 30, 2009, were abstracted. The NIS is a longitudinal hospital inpatient database included in the Healthcare Cost and Utilization Project (HCUP) family, created by the Agency for Healthcare Research and Quality through a federal-state partnership [8]. The database includes discharge abstracts from 8 million hospital stays and incorporates patient and hospital information, including patients covered by Medicare, Medicaid, private insurance, and other insurance types.

Each discharge includes up to 15 inpatient diagnoses and procedures per hospitalization. All procedures and diagnoses are coded using the* International Classification of Disease, 9th revision, Clinical Modification* (ICD-9-CM). Available patient and sociodemographic characteristics include gender, race, age, expected source of payment, and outcome (in-hospital mortality), as well as hospital information (unique hospital identifier, hospital location, and hospital volume). Patients' socioeconomic status was evaluated using a proxy income, defined by county-specific ZIP code according to the US Census. In accordance with institutional policy with regard to publicly available data, this study was exempt from institutional review board approval.

### 2.2. Study Population

A total of 8 major surgical oncological procedures were selected for the evaluation of postoperative pneumonia: colectomy, cystectomy, esophagectomy, gastrectomy, hysterectomy, pneumonectomy, pancreatectomy, and prostatectomy. Analyses were restricted to cancer diagnoses only. Relying on specific ICD-9-CM procedure codes, each surgical procedure was assessed independently.

### 2.3. Primary Outcome

Primary outcome was pneumonia in the postoperative timeframe, defined according to previous criteria (ICD-9-CM 480–487) [9]. These include viral pneumonia (ICD-9-CM-480), pneumococcal pneumonia (ICD-9-CM-481), other bacterial pneumonia (ICD-9-CM-482), pneumonia due to other specified organisms (ICD-9-CM-483), pneumonia in infectious diseases classified elsewhere (ICD-9-CM-484), bronchopneumonia organism unspecified (ICD-9-CM-485), pneumonia organism unspecified (ICD-9-CM-486), and influenza (ICD-9-CM-487).

### 2.4. Patient and Hospital Characteristics

Available independent variables for analyses included patient age at hospitalization, race, sex, insurance status, baseline comorbidities, and median household income by ZIP code, as well as hospital location. Information on race was categorized as White, Black, Hispanic, other (Asian or Pacific Islander, Native American), or unknown. Insurance status was classified based on the expected primary payer and included Medicare, Medicaid, private insurance, and other insurance types including those who were uninsured. Patient age was considered as a continuous variable. Baseline comorbidities were determined using a Charlson comorbidity Index-derived score [10], adapted by Deyo et al. [11].

To estimate patient income levels, we relied on the median household income of the patient's ZIP code of residence, which was derived from the US Census. Four categories were available within the database: [1] <$25,000, [2] $25,000–34,999, [3] $35,000–44,999, and [4] ≥$45,000.

Hospital characteristics including United States Census Bureau region (Northeast, Midwest, South, and West), population density (rural versus urban), and teaching status were obtained from the American Hospital Association Annual Survey of Hospitals. A hospital was considered a teaching institution if it had an American Medical Association-approved residency program, was a member of the Council of Teaching Hospitals, or had a ratio of 0.25 or higher of full-time equivalent interns and residents to nonnursing home beds [12]. Annual hospital volume represents the number of procedures done by each participating institution during each study calendar year and was calculated independently for each of the eight procedures. Patients were divided according to hospital volume quartiles, categorized as very low, low, high, and very high.

### 2.5. Statistical Analysis

Data distribution was adjusted according to the provided NIS population weights in order to render estimates more accurate nationally. All analyses were performed on the weighted population.

First, descriptive statistics were generated on frequencies and proportions for categorical variables (gender, race, insurance status, median ZIP code household income, CCI, annual hospital volume, hospital location, hospital region, and hospital teaching status) and stratified according to pneumonia occurrence. Means, medians, and interquartile ranges were reported for continuous variables (age, year of surgery). Chi-square, independent *t* tests, and Kruskal-Wallis tests were used to compare the statistical significance of differences within categorical and continuous variables.

Second, temporal trends in rates were analyzed by the estimated annual percentage change (EAPC), which uses the least squares linear regression methodology as suggested by Anderson et al. [13].

Third, multivariable logistic regression analyses were fitted to predict pneumonia following MCS. Year of surgery, age, race, baseline CCI, median ZIP code household income, hospital location, hospital region, and hospital teaching status were considered as covariates. Fourth, separate models were fitted with mortality as the outcome and pneumonia as an independent variable. Finally, to adjust clustering within hospitals, multivariable logistic regression models were fitted with generalized estimating equations [14].

All statistical analyses were performed using the R statistical package (R Foundation for Statistical Computing, Vienna, Austria), with a two-sided significance level set at *P* < 0.05.

## 3. Results

### 3.1. Baseline Descriptors

A weighted estimate of 2,508,916 patients underwent one of the 8 chosen procedures. Baseline sociodemographic characteristics in the entire cohort are described in Table 1. Average patient age was 65.9 (median 66.0). Most patients were male (60.3%) and Caucasian (60.8%). Almost all payment forms were private (42.2%) and Medicare (50.5%). Procedures were done predominantly in urban hospitals (89.3%) and slightly more than half (54.7%) were performed at teaching hospitals.

### 3.2. Pneumonia after MCS: Rates and Trends

**Figure 1:**
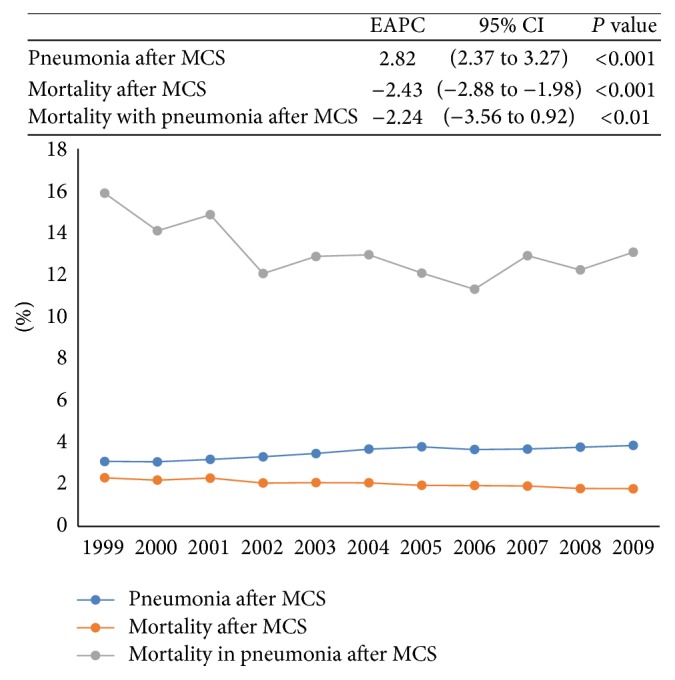
Pneumonia and mortality with pneumonia in major cancer surgery (MCS) patients between 1999 and 2009 in the United States. EAPC: estimated annual percentage change and CI: confidence interval.

Between 1999 and 2009, 3.5% of patients undergoing MCS had postoperative pneumonia.  Figure 1 describes temporal trends. The estimated annual percent change (EAPC) of pneumonia was +2.8% over the course of the study (95% CI [2.4 to 3.3], *P* < 0.001). During the same period, the EAPC of mortality in patients undergoing MCS was −2.4% (95% CI [−2.9 to −2.0], *P* < 0.001), while the EAPC of mortality in patients undergoing MCS who developed pneumonia was −2.2% (95% CI [−3.6 to 0.9], *P* = 0.01).

### 3.3. Patient Characteristics and Pneumonia after MCS

Multivariable logistic regression analyses predicting the occurrence of pneumonia after MCS are reported in Table 2. Increased odds of developing pneumonia were associated with increasing age (OR: 1.024, *P* < 0.001), male patients (female OR: 0.919, *P* < 0.001), and comorbidities (CCI 1–3 versus 0 OR: 2.084–2.095, all *P* < 0.001). All nonprivate patients had significantly increased risk of pneumonia diagnosis (versus private OR: 1.3–2.4, all *P* < 0.001). Median household income also achieved independent predictor status, with each stepwise increase in income brackets showing a reduction in the odds of pneumonia, with the highest bracket showing 19% less likely of pneumonia than the lowest bracket (OR: 0.81, *P* < 0.001).

### 3.4. Hospital Characteristics and Pneumonia after MCS

Hospital characteristics associated with pneumonia after MCS are also shown in Table 2. As hospital volume increased, pneumonia rates decreased (very high, high, or low versus very low OR: 0.721–0.923, all *P* < 0.001). Urban hospital location was associated with significantly greater odds of pneumonia relative to rural areas (OR: 1.182, *P* < 0.001). Hospital region was a significant predictor: the Midwest and West regions were significantly associated with lower rates of pneumonia compared to the Northeast (respective OR: 0.937 and 0.839, both *P* ≤ 0.009). Teaching hospital status also demonstrated less pneumonia diagnoses (OR: 0.875, *P* < 0.001).

### 3.5. Mortality and Pneumonia after MCS

Mortality varied according to cancer surgery, as shown in Table 3. After multivariable analyses adjusting patient and hospital characteristics, patients with pneumonia after MCS showed a 6.3-fold increase in mortality relative to patients without pneumonia. Even though hysterectomy (0.3%) and prostatectomy (0.1%) showed the lowest rates of pneumonia after MCS, the effect of pneumonia on mortality was the highest for these procedures: hysterectomy (OR: 13.2, *P* < 0.001) and prostatectomy (OR: 8.7, *P* < 0.001). Conversely, the procedures associated with the highest prevalence of pneumonia after MCS concurrently had the smallest effect on mortality: esophagectomy (OR: 2.57, *P* < 0.001), pancreatectomy (OR: 2.74, *P* < 0.001), and gastrectomy (2.94, *P* < 0.001).

## 4. Discussion

Postoperative pneumonia is one of the biggest causes of surgical morbidity, being responsible for approximately 25% of postoperative deaths occurring within six days after surgery [3]. Although validated regional and national guidelines for the management of pneumonia exist [6, 7], their efficacy in the postoperative patient cohort has not been fully studied. Given that major oncologic procedures are known to predispose to an especially high risk of pneumonia [4, 5], we sought to analyze population-level trends in the prevalence and mortality of pneumonia following MCS in the past decade. Additionally, by evaluating patient and hospital factors, we attempted to identify areas for improvement in the management of pneumonia after MCS and specific oncologic surgeries that would most benefit from these.

Our results raise numerous noteworthy findings. First, while the overall rate of in-hospital pneumonia following MCS was 3.5%, a figure similar to previous reports where rates of 0.8–6.2% were seen after different types of cancer surgery [15–18], its prevalence increased by 29.7% between 1999 and 2009. Yet, the EAPC of mortality of patients who developed pneumonia after MCS was −2.2% (95% CI: −3.6 to 0.9), similar to the EAPC of mortality of all MCS patients in the same period. This finding of increasing prevalence and decreasing mortality suggests that pneumonia is becoming better recognized in the postoperative cancer surgery patient and confirms the value of adhering to management guidelines that have been proven to improve outcomes [19–21].

We were also able to identify patient and hospital characteristics associated with a higher risk of pneumonia after MCS. As expected, older, sicker, and male patients were at higher odds of developing pneumonia [17, 22–24]. Interestingly, nonprivate insurance was an independent predictor for pneumonia, with Medicaid patients at greatest risk, followed by Medicare and other forms of payment (i.e., uninsured, self-payment, and no payment). Previous reports have suggested that insurance-related disparities may lead to reduced access to high-quality care [25]. These individuals are also less likely to be treated at academic, high-volume centers [26], which have superior facilities and higher staff/patient ratios, thereby offering an explanation as to why they are at greater risk of in-hospital complications [27].

Regression analysis of hospital characteristics showed that individuals treated at teaching hospitals were 12% less likely to develop post-MCS pneumonia. Further, patients treated at very high, high, and low volume institutions were 28%, 17%, and 8% less likely to develop postoperative pneumonia compared to very low volume hospitals, respectively. This may be a result of selective referral and the subsequent selection of healthier patients, improved compliance to guidelines at higher volume centers, or a combination of the two [28, 29]. Our finding that patients managed at urban hospitals had a higher risk of postoperative pneumonia may be explained by an increased likelihood of these individuals being exposed to more virulent nosocomial pathogens [30].

Finally, we attempted to corroborate the relationship between pneumonia and mortality after surgical procedures [31, 32]. On average, patients who developed pneumonia after MCS had a greater than sixfold risk of mortality compared to those who did not, with the highest pneumonia-associated death rates seen in esophagectomy (15.8%), gastrectomy (14.5%), and pancreatectomy (14.6%). However, the specific effect of pneumonia on mortality was the lowest with these three procedures (OR: 2.6–2.9, all *P* < 0.001), suggesting that the inherent morbidity of these higher-risk surgeries and other competing causes of mortality may lessen the physiologic insult that pneumonia places in this upper gastrointestinal cancer surgery cohort. This is supported by the finding that pneumonia had the greatest impact on mortality after hysterectomy and prostatectomy (OR: 8.7–13.2, *P* < 0.001), which are arguably far less morbid procedures.

It is important to appreciate that several specific limitations apply to our study in addition to those that are inherent to all retrospective observational analyses. It was not possible to know how each case of pneumonia was ascertained, since we relied on ICD-9-CM diagnostic codes from the diagnosis fields in the NIS. It may also be possible that certain patients had pneumonia preoperatively, although most cancer surgery patients would have likely undergone preoperative chest radiographs. Another related limitation was the lack of temporal information on pneumonias, as the exact timing could have ensured a particular impact on mortality. Moreover, we were unable to control for certain risk factors, such as the duration of surgery and of postoperative ventilation, which may have placed patients at risk of a ventilator-associated pneumonia (VAP). By means of a large, population-based analysis, however, we were able to attenuate these limitations significantly to provide reliable evidence regarding the objectives of this study. Finally, there are certain limitations inherent to the use of administrative data in the context of healthcare associated infections [33]. We addressed them by enlarging the pneumonia spectrum as much as possible, in order to reduce subtle variations in classification terms, such as “other bacterial pneumonia” versus “bronchopneumonia organism unspecified.” We also cross-validated our results with nonadministrative data, and they yielded similar rates of pneumonia during inpatient stays.

To our knowledge, this is the first study to evaluate the trends and patterns of pneumonia after multiple types of major cancer surgeries. It is encouraging that the overall awareness for this potentially fatal postoperative complication has risen, thus potentially averting unnecessary mortality. Furthermore, our results are a clear mandate for the referral of these challenging oncological procedures to experienced, high-volume centers.

## Figures and Tables

**Table 1:** Weighted descriptive characteristics of 2,508,916 patients > 18 years old undergoing major cancer surgery, Nationwide Inpatient Sample, 1999–2009.

Variables	Overall (%)	Without pneumonia (%)	With pneumonia (%)	*P*
*Weighted number of patients*	2508916	2421049 (96.5)	87867 (3.5)	**—**

*Age (years)*				<0.001^†^
Mean (SD)	65.9 (11.7)	65.8 (11.6)	70.7 (12.0)	
Median (IQR)	66.0 (58,74)	66.0 (58,74)	72.0 (63,79)	

*Gender*				<0.001
Male	1511360 (60.3)	1460516 (60.4)	50844 (57.9)	
Female	993705 (39.7)	956692 (39.6)	37013 (42.1)	

*Race*				<0.001
Caucasian	1525021 (60.8)	1470817 (60.8)	54204 (61.7)	
Black	177986 (7.1)	171819 (7.1)	6167 (7.0)	
Hispanic	98351 (3.9)	95249 (3.9)	3282 (3.7)	
Other	93041 (3.7)	90005 (3.7)	3036 (3.5)	
Unknown	614337 (24.5)	593159 (24.5)	21178 (24.1)	

*CCI*				<0.001
0	1566723 (62.4)	1530952 (63.2)	35771 (40.7)	
1	623985 (24.9)	590474 (24.4)	33511 (38.1)	
2	127538 (5.1)	120123 (5.0)	7415 (8.4)	
≥3	190671 (7.6)	179501 (7.4)	11170 (12.7)	

*Insurance status*				<0.001
Private	1057919 (42.2)	1036830 (42.8)	21089 (24.0)	
Medicaid	80666 (3.2)	76452 (3.2)	4214 (4.8)	
Medicare	1265919 (50.5)	1206553 (49.8)	59366 (67.6)	
Other	104412 (4.2)	101214 (4.2)	3198 (3.6)	

*Median household income by ZIP code*				<0.001
** **1–24,999	369797 (14.7)	353335 (14.6)	16462 (18.7)	
25,000–34,999	596203 (23.8)	573382 (23.7)	22821 (26.0)	
35,000–44,999	646868 (25.8)	624454 (25.8)	22414 (25.5)	
45,000+	842375 (33.6)	817918 (33.8)	24457 (27.8)	
Unknown	53672 (2.1)	51959 (2.1)	1713 (1.9)	

*Annual hospital volume*				<0.001
Very low	591676 (23.6)	566674 (23.4)	25002 (28.5)	
Low	640229 (25.5)	615916 (25.4)	24313 (27.7)	
High	636482 (25.4)	615423 (25.4)	21059 (24.0)	
Very high	640531 (25.5)	623037 (25.7)	17494 (19.9)	

*Hospital location*				<0.001
Rural	268348 (10.7)	257549 (10.6)	10799 (12.3)	
Urban	2239651 (89.3)	2162616 (89.4)	77035 (87.7)	

*Hospital region*				<0.001
Northeast	526593 (21.0)	507937 (21.0)	18656 (21.2)	
Midwest	608987 (24.3)	587593 (24.3)	21394 (24.3)	
South	882566 (35.2)	849637 (35.1)	32929 (37.5)	
West	490770 (19.6)	475882 (19.7)	14888 (16.9)	

*Hospital teaching status*				<0.001
Nonteaching	1135065 (45.3)	1088497 (45.0)	46568 (53.0)	
Teaching	1372936 (54.7)	1331669 (55.0)	41267 (47.0)	

^†^Mann-Whitney test.

CCI: Charlson comorbidity index, SD: standard deviation, and IQR: interquartile range.

**Table 2:** Multivariable logistic regression analysis predicting the occurrence of pneumonia following major cancer surgery, Nationwide Inpatient Sample, 1999–2009.

Variables	OR (95% CI)	*P*
*Age*	1.024 (1.019–1.03)	<0.001

*Gender*		
Male	1.0 (ref.)	
Female	0.919 (0.89–0.948)	<0.001

*Race*		
Caucasian	1.0 (ref.)	
Black	0.996 (0.937–1.059)	0.894
Hispanic	0.911 (0.839–0.988)	0.024
Other	0.994 (0.914–1.081)	0.895
Unknown	1.038 (0.999–1.078)	0.059

*CCI*		
0	1.0 (ref.)	
1	2.084 (2.014–2.158)	<0.001
2	2.095 (1.976–2.221)	<0.001
≥3	2.087 (1.985–2.195)	<0.001

*Year of surgery*	1.024 (1.019–1.03)	<0.001

*Insurance status*		
Private	1.0 (ref.)	
Medicaid	2.376 (2.196–2.569)	<0.001
Medicare	1.333 (1.272–1.396)	<0.001
Other	1.412 (1.297–1.538)	<0.001

*Median household income by ZIP code*		
1–24,999	1.0 (ref.)	
25,000–34,999	0.909 (0.867–0.953)	<0.001
35,000–44,999	0.873 (0.832–0.916)	<0.001
45,000+	0.811 (0.772–0.851)	<0.001
N/A	0.821 (0.733–0.919)	<0.001

*Annual hospital volume*		
Very low	1.0 (ref.)	
Low	0.923 (0.885–0.963)	<0.001
High	0.829 (0.792–0.868)	<0.001
Very high	0.721 (0.685–0.758)	<0.001

*Hospital location*		
Rural	1.0 (ref.)	
Urban	1.182 (1.122–1.244)	<0.001

*Hospital region*		
Northeast	1.0 (ref.)	
Midwest	0.937 (0.893–0.984)	0.009
South	0.983 (0.941–1.026)	0.427
West	0.839 (0.798–0.883)	<0.001

*Hospital teaching status*		
Nonteaching	1.0 (ref.)	
Teaching	0.875 (0.845–0.907)	<0.001

OR: odds ratio, CI: confidence interval, ref.: referent category, and CCI: Charlson comorbidity index.

**Table 3:** Multivariable logistic regression analysis after fitting with generalized estimating equation (GEE) and covariables (age, gender, race, CCI, insurance status, median household income by ZIP code, annual hospital volume, hospital location, hospital region, and hospital teaching status) for predicting mortality in the context of pneumonia following MCS, Nationwide Inpatient Sample, 1999–2009.

Procedure type	Mortality				*P* value
	Overall (%)	Without pneumonia (%)	With pneumonia (%)	OR (95% CI)	
Overall	51312 (2.0)	39865 (1.6)	11448 (13.1)	6.267 (5.994–6.609)	<0.001
Colectomy	28651 (3.1)	23418 (2.6)	5232 (13.5)	4.45 (4.132–4.793)	<0.001
Cystectomy	1993 (2.5)	1711 (2.2)	283 (10.6)	3.907 (2.841–5.373)	<0.001
Esophagectomy	1264 (7.2)	894 (5.9)	370 (15.8)	2.569 (1.877–3.516)	<0.001
Gastrectomy	4677 (5.7)	3709 (4.9)	968 (14.5)	2.944 (2.480–3.496)	<0.001
Hysterectomy	906 (0.4)	782 (0.3)	124 (5.7)	13.17 (8.213–21.118)	<0.001
Lung	10572 (2.9)	6498 (1.9)	4075 (13.4)	7.371 (6.697–8.113)	<0.001
Pancreatectomy	2762 (4.9)	2384 (4.4)	377 (14.6)	2.735 (2.083–3.593)	<0.001
Prostatectomy	487 (0.1)	469 (0.1)	19 (0.9)	8.727 (3.105–24.528)	<0.001

OR: odds ratio and CI: confidence interval.
